# Radical Collaboration: Reimagining Cancer Team Science

**DOI:** 10.1158/2159-8290.CD-23-1496

**Published:** 2024-04-04

**Authors:** Jesse S. Boehm, Tyler Jacks

**Affiliations:** 1 Break *Through* Cancer, Cambridge, Massachusetts.; 2 David H. Koch Institute for Integrative Cancer Research, Massachusetts Institute of Technology, Cambridge, Massachusetts.

## Abstract

Here, we define a future of cancer team science adopting “radical collaboration”—in which six “Hallmarks of Cancer Collaboration” are utilized to propel cancer teams to reach new levels of productivity and impact in the modern era. This commentary establishes a playbook for cancer team science that can be readily adopted by others.

## INTRODUCTION

Following the 2007 global financial crisis, funds from the American Recovery and Reinvestment Act bolstered an ambitious, controversial project that required scientists to stretch outside their comfort zones and work collaboratively. By 2018, that project, the Cancer Genome Atlas (TCGA), had produced over 2.5 petabytes of data on 33 tumor types and transformed our understanding of cancer. TCGA involved thousands of scientists across 20 institutions and presented a new template for large-scale structured collaboration in the cancer community.

Emerging from the COVID-19 pandemic, our community once again has a rare opportunity to hasten progress toward its most ambitious goals by working collaboratively. But to do so requires a radically new approach. Although discovery science and individual accomplishments typically provide the starting point for future clinical breakthroughs, in isolation, innovations are not enough. To maximize our ability to bring impactful treatments to patients as rapidly as possible, the community must leverage new structural approaches that reform how teams of cancer researchers coalesce, function, and thrive in the modern era.

In this commentary, we attempt to identify the essential ingredients underpinning “next-generation” cancer team science experiments under way in the United States and abroad. In doing so, we suggest a first draft of six “Hallmarks of Cancer Collaboration.” Following the structure of Hanahan and Weinberg's seminal work presenting the Hallmarks of Cancer (1, 2), we hope the Hallmarks of Cancer Collaboration will lay a tangible foundation from which others might study the sociological and operational aspects of how to improve the efficiency and impact of modern cancer teams.

## BACKGROUND

This is an unprecedented moment for oncology. After decades of steady progress, now nearly 30% of patients with cancer benefit from recent medicines developed from our improved understanding of the molecular biology and genetics of cancer (3). And we have only just begun: Unparalleled breakthroughs in single-cell and spatial genomics, machine learning and data science, and novel therapeutic modalities provide fundamentally new opportunities for impact. Within the next five years, national and international cancer data generation and aggregation initiatives will achieve the necessary scale and precision to drive diagnosis and intervention earlier than ever before.

But, unless the cancer community takes fundamentally new steps to work together, competition and fragmentation threaten the pace of progress. Given the magnitude of the challenge and the diverse skills needed to intercept and cure the most difficult cancers, we believe we must harness the same team-oriented, mission-driven ambition that propelled the rapid success of COVID-19 research (4) and vaccines. In fact, the same number of Americans that died each day at the height of the pandemic roughly equates to the number that continue to die each day of cancer. We anticipate that the degree of systemic restructuring needed to urgently tackle COVID-19 and cancer is likely to be similar.

The structure of the current scientific enterprise in the United States is, surprisingly, only about 80 years old. It was largely established after World War II based on recommendations of engineer and science administrator Vannevar Bush's 1945 “Science, the Endless Frontier” report (5). Bush, then Director of the Office of Scientific Research and Development under President Franklin Delano Roosevelt, called for the federal government to fund basic research at individual universities, colleges, and research institutes, and to invest in the training and development of individual scientists so as to make progress in the war against disease. We believe the next 80 years of cancer research will require augmentation of that structure—changes to help propel deeply collaborative, explicitly goal-oriented team-based inquiries.

Of course, the historical seeds of today's acceleration toward new collaborative models began far before the pandemic. They were sowed over decades of pioneering activities driven in the United States by the NCI, beginning with the NIH Program Project Grant mechanism (PO1) and followed by the Specialized Programs of Research Excellence (SPORE) program. In the 1990s and 2000s, consortia models began connecting broader networks of investigators to flesh out emerging fields and establish common knowledge, standards, and reagents (e.g., Mouse Models of Human Cancer Consortium and Early Detection Research Network). The National Human Genome Research Institute's pioneering Human Genome Project provided an indelible template for team-based biomedical science, as did the NCI-led TCGA (https://www.cancer.gov/ccg/research/genome-sequencing/tcga/history).

## A MODERN LANDSCAPE

Today, in 2024, large-scale collaborative endeavors have adopted many of the components of these earlier NCI-led activities. Although we do not have sufficient space to be exhaustive, here we provide several examples:

– The success of TCGA stimulated numerous team-based genomics collaborative efforts, including AACR's Project GENIE (3) and the Pan-Cancer Analysis of Whole Genomes effort (6), driven by the International Cancer Genomics Consortium.
– Beyond genomics, the Cancer Moonshot, launched by the Obama–Biden Administration in 2016 and reignited in 2022 by the Biden–Harris Administration, reframed key needs and united academia, biopharma, tech, nonprofit, and governmental organizations to promote team-based, systems-level reform.
– The Cancer Research UK (CRUK) Cancer Grand Challenges program, launched in 2015 and then partnered with the NCI in 2020, provides transformative resources to international, interdisciplinary teams tackling provocative questions to some of cancer's most complex problems. Cancer Research Horizons is CRUK's innovation engine that manages licensing, commercialization, and biopharma partnerships to provide a translational route for innovations emerging from the program.
– The Parker Institute for Cancer Immunotherapy (PICI) invests in catalytic projects and team-based centers focused on stimulating understanding of cancer immunity and immunotherapy and has developed an expansive network of over 50 biopharma partnerships.
– The Mark Foundation for Cancer Research utilizes a nimble, high-impact approach to bridge the gap between bench and bedside in cancer research, including team-based centers. Multiple activities are in partnership with other foundations or biopharma partners.
– Stand Up to Cancer (SU2C), with partners such as AACR, CRUK, and the Lustgarten Foundation, funds “Dream Teams” of cancer researchers from different institutions, stimulated by energy and investment from the general public and the entertainment industry with multiple partners and biopharma collaborations.
– Our organization, Break *Through* Cancer, works with foundation and biopharma partners to create virtual shared laboratories across institutions, called TeamLabs, that centrally manage resources to tackle ambitious cancer challenges. Each TeamLab shares data and discoveries in real time.

In addition to cancer-focused efforts, technology-focused biomedical efforts are helping to shape the future of cancer team science in profoundly exciting ways:

– The Chan Zuckerberg Initiative (CZI) leads multiple team-based open science efforts to create unrestricted technologies and resources for the scientific community and played a key role in the launch of the international Human Cell Atlas (7).
– The Advanced Research Projects Agency for Health (ARPA-H) aims to bring a DARPA (Defense Advanced Research Projects Agency)-like team-science model forward, in which program managers lead streamlined, high-impact efforts leveraging technological breakthroughs.

With these and other efforts in aggregate, we would estimate conservatively that US $500M–$1B is being invested annually in team-based approaches to solve challenges in cancer in academia alone. Importantly, this commentary should cast no doubt that individual investigator-led basic research remains a fundamental source of insights and technologies that underpin the field's progress. Continued support of this style of cancer science must continue unabated.

Over the past 20 years, across disciplines from particle physics to social sciences, teams are increasingly seen as a central force in the process of knowledge creation (8, 9). Today, we imagine the average American assumes that cancer researchers work collaboratively and share data. Even within oncology—a field with enormous investment and high public expectations—it would be reasonable to assume that we have a clear, widely adopted playbook of best practices for cancer team science.

Unfortunately, no such playbook exists.

**Figure 1.**
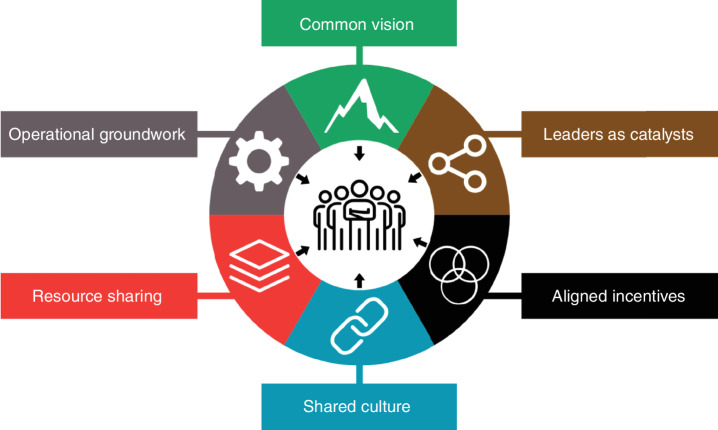
The Hallmarks of Cancer Collaboration. Based on Hanahan and Weinberg's seminal “Hallmarks of Cancer” (1), this illustration proposes six Hallmarks of Cancer Collaboration for cancer team science in the modern era.

Here, we begin to outline those best practices by articulating the “Hallmarks of Cancer Collaboration” (Fig. 1): six pillars that we believe are the ingredients to propel research teams to achieve new levels of productivity and impact in the modern era of cancer science.

Together, these pillars create what we call *radical collaboration* (Box 1). To the best of our understanding, the future of cancer team science cannot be fully realized without radical collaboration-driven structural reform that fully explores and adopts each of the following hallmarks.

Box 1. **DEFINING RADICAL COLLABORATION**

For maximal ontological clarity in this commentary, we distinguish three terms:

***Collaboration*** is a general term, typically including any form of interaction among multiple researchers receiving joint funding.

***Team science*** is a larger number of investigators working together toward a common goal, typically leveraging organizational structures that currently exist.

***Radical collaboration*** refers to a new form of collaborative team science that champions the six Hallmarks of Cancer Collaboration: common vision, leaders as catalysts, aligned incentives, shared culture, resource sharing, and operational groundwork.

## HALLMARKS OF CANCER COLLABORATION

Each key component below is described in detail, illustrated with examples to demonstrate its functional importance, and linked to strategies by which ongoing team-science initiatives are testing the component in real-world settings.

### Common Vision

A NASA-led team-science mission to redirect a 492-foot diameter asteroid located 7 million miles from Earth; the Biden Cancer Moonshot's ambition to enable easy access to one's personal health information for all patients with cancer; or Chan Zuckerberg Biohub New York's plans to bioengineer immune cells to detect and treat problems before they spark untreatable disease. Each of these real-world examples demonstrates a bold, clear, and urgent vision for a goal that would be plausibly impossible to achieve by one laboratory or one institution. Common vision is an essential driving force for radical collaboration.

We believe such a vision requires a slow formation process of deep engagement, codeveloped by a team of stakeholders (including researchers in academia and biopharma, data scientists and computational biologists, patients and policymakers) who subsequently see the shared vision as their own, because it was authentically the product of joint conception. All too often, we see a single stakeholder craft a vision or draft a research plan unilaterally and “invite” others to join the team. This approach to a project's vision can produce unequal commitment. In other scenarios, a funder may desire “more collaboration” without first defining a common vision. Again, this can lead to a mismatch between the goal and the team structure.

Importantly, team science benefits from involving investigators with different research backgrounds from the very conception of the common vision. Innovation occurs at the intersection of new ideas, as people with different experiences and backgrounds are not tethered to “the status quo” of the field. To enable this interdisciplinary collaboration, investigators should learn the language of each other's fields, or have an expert on the team who can translate between and among specialties.

Finally, an important, yet seemingly paradoxical lesson from the DARPA model is that the audacity of a team's vision is directly proportional to the passion and commitment that members of the team bring to bear on the challenge. In other words, the more ambitious and potentially “risky” an effort appears to be, the less likely a traditional funder is to fund it, yet the more likely it is to sustain engagement and have a good chance at succeeding. In our experience, “lower-risk” next steps or incremental efforts end up failing more frequently because scientists are less invested in them. Ambitious projects with a clear common vision, proposed by investigators capable of extraordinary achievement, should be compelling to team science funders.

### Leaders as Catalysts

Academic cancer research is largely a hierarchical enterprise. This bestows certain advantages, such as clear accountability. However, the overreliance on a hierarchical system can also breed disadvantages, such as marginalizing voices from women and scientists from underrepresented backgrounds. In addition, current systems of cancer incentives (see next hallmark) may over-reward formal leadership (named, senior leaders given institutional authority) and under-reward informal leadership (junior investigators that rise to a challenge or lead without formal authority). Cancer team science needs a diversity of membership to achieve its potential.

Clear and effective leadership for radical collaboration requires a small number of highly committed, highly effective leaders who are well-trained not only in biological, clinical, and computational arenas but also in the sociology of bringing teams together, sustaining engagement, and driving productivity. In this way, leaders of team science in an era of radical collaboration must be catalysts, keeping the work, not themselves, at the center. After all, great ideas can come from anyone, and conditions for creativity and passion to flourish must be maintained.

For example, PICI Centers have directors with the flexibility to allocate resources and thoughtfully engage a diversity of leaders to participate. At Break *Through* Cancer, TeamLabs are led by “culture carriers” who emerge organically at the start of an effort, representing the labs that show the most on-the-ground commitment to working collaboratively.

Very importantly, multiple organizations are making deep and sustained commitments to recruiting, developing, and retaining leaders and scientists from underrepresented ethnic and socioeconomic backgrounds. Diversity among personnel provides valuable diversity of thoughts, ideas, and processes, leading to robust, richer development and implementation of the team's common vision, and should be embraced by all cancer team science efforts.

### Aligned Incentives

Achieving the lofty ambitions of cancer team science in the modern era is made more challenging by incentive structures that reward individual contributions. Established systems value published papers over products and drive individuals toward personal accolades to maximize chances for academic promotion (10). They may also accidentally reduce the emphasis on contributions from data scientists and engineers. Additionally, current incentive systems reinforce a culture that values formal leadership over leaders as catalysts.

The incentive to participate in cancer team science should be the opportunity to contribute meaningfully toward life-saving cures for cancer in a manner that is impossible to achieve individually. Team members can be incentivized by participation in vibrant intellectual interchange; the opportunity to work alongside distinguished scientists and clinical investigators from multiple disciplines and institutions, including highly trained experts at biopharma companies; access to funding and important data sets; and opportunities for mentorship, connections, and career advancement. Leaders, acting as catalysts, can highlight these incentives by emphasizing the common vision, and they should consistently monitor whether such conditions are met within the team.

Funders can take active roles in supporting the career development of junior colleagues, with particular attention to women and investigators from underrepresented backgrounds. Career development committees, such as those instituted for large-scale particle physics collaborations, can help identify emerging talent, distribute speakership invitations and prize nominations in a meritocratic manner, and support individual promotion cases. Funders, institutional leaders, and other stakeholders should volunteer to write detailed letters of support for individuals making outstanding contributions to team projects. Finally, team-based authorship models need to be uniformly implemented, such as those adopted by NCI-funded teams in the post-TCGA era (6).

Incentives around intellectual property represent another complex area when multiple institutions are involved. Here, we suggest structural policy changes, whether from funders or governmental agencies, are needed to ensure policies fit a radical multi-institutional future of cancer team science. Deep relationships between business development offices, funders and PIs can ensure that decisions on filing, licensing, and commercialization strategies benefit (a) patients and (b) sustain the mission and incentives of the team, while (c) providing financial benefit to individual institutions. We recognize the complex realities of this schema, yet it is essential. Funders that bring transformative levels of funding (>$100M or more) might be able to help ease the tension among those priorities.

### Shared Culture

Cancer teams are communities. Like any community, scientific or otherwise, cohesiveness directly relates to the degree to which a shared cultural bond is established and maintained. This bond is fostered by clearly articulated values and consistently upheld and reinforced principles. We imagine a radically collaborative future of cancer team science built on a foundation of shared culture and a shared identity that transcends individuals, institutions, and companies.

Cancer teams should aspire to create an environment of directness, urgency, honesty, and authenticity. Friction should not be avoided, as disagreements over strategy and execution planning are natural in any fast-paced, goal-oriented arena. However, careful diagnosis of the sources of the friction—personal, financial, or scientific—and subsequent resolution requires a shared commitment to depersonalize conflict and keep the work at the center.

At the Mark Foundation, the level of trust and potential for collaboration is part of the evaluation process, assessed during live interviews. At PICI, in-person retreats twice per year—to which families of scientists are also invited—form meaningful, personal bonds between scientists and encourage cross-team cohesiveness. At these meetings, unpublished data and ideas are discussed, a feeling of openness is fostered, and relationships are created such that each scientist emerges with an expanded network.

At ARPA-H, the culture is “administratively and scientifically nimble” and investigators are encouraged to take risks. At Break *Through* Cancer, TeamLab members participate in in-person workshops, share data and resources, and are propelled by catalytic leaders. Multiple organizations spend time and resources on team-patient interactions, which refresh a sense of purpose for all those involved.

### Resource Sharing

Productive communities share and exchange resources sans friction or boundaries. For productive cancer teams, this includes sharing ideas, data and algorithms, biospecimens, dollars, and even personnel. Although funders may assume that barriers to sharing are based on attitudes, we experience barriers to sharing in the form of structures imposed by institutions and institutional review boards, such as material transfer agreements, human subjects obligations, conflicts of interest, etc. At best, these institutional structures require significant resources and commitment to overcome. At worst, they create regular and repeated sources of friction and can effectively paralyze collaboration.

Progress is being made. In 2017, updates to the Common Rule (11) streamlined key challenges, including establishing single IRBs for multi-institutional studies in the United States beginning in 2020 and allowing the use of broad-based consents for unspecified, future secondary uses of collected biospecimens. The 2023 NIH Data Management and Sharing Policy (https://grants.nih.gov/grants/guide/notice-files/NOT-OD-21-013.html) prompts cancer researchers receiving federal grants to think proactively about data exchange.

Transformative funders of cancer team science can help improve resource sharing. In the case of data, PICI and the Biden Cancer Moonshot have made long-standing investments in supporting the collection and aggregation of data to freely share among teams; the NCI developed the Genomic Data Commons to foster genomic data aggregation and sharing; and the Chan Zuckerberg CELL by GENE Discover tool leverages over 1,159 single-cell data sets for collaboration. Personnel and dollars are also “shared.” For example, CRUK encourages scientists to move between teams, even internationally, with trainees working under co-PIs, and PIs taking sabbaticals in other labs.

We provide two cautionary notes. First, sharing for the sake of sharing can be a downfall. Sharing should be a solution in pursuit of the common vision. Otherwise, resources will be consumed without scientific impact. Second, sharing is expensive. Some initiatives consume hundreds of thousands or even millions of dollars to make data authentically findable, accessible, interpretable, and reusable (12). Effective sharing will often require funders to provide resources to support data managers, engineers, tech transfer staff, clinical research coordinators, and project managers, and to alleviate the tension, if it exists, between commercial interest and the free flow of data. Investments in resource sharing should be viewed as an exciting driver of scientific impact, not a tax.

### Operational Groundwork

Our final proposed Hallmark of Cancer Collaboration is the foundation upon which all of the others stand—and it is typically overlooked. To make the future of cancer team science radically different from the past, funders, scientists, and institutions must recognize the importance of professional management: people, tools, structures, and policies that enable the scientific enterprise to function.

As a field, we must recruit, retain, and promote management experts who are well-versed in the disciplines of business consulting, agile-based management practices, efficiency-driven thinking, and human-centered design. For optimal success, these experts should work alongside scientific leaders to drive programs forward.

ARPA-H efforts are supported by experts in security, legal, and contracting issues, finance, human resources, and communications. Institutional Alliance Managers and project managers embedded at partner institutions work with dedicated TeamLab liaisons used by Break *Through* Cancer. PICI funds and supports full-time, embedded technology transfer employees at each supporting institution.

Finally, investments in new types of non-tenure track Research Scientists who sit at the intersection of scientific innovation and community building are emerging as extremely important. Our “Break *Through* Cancer Scientists” program is one such experiment in this area; these scientists receive salary support to provide career stability as they help maximize and catalyze the impact of an entire TeamLab. CZI's Imaging Scientists grants invest in experts who work at the intersection of biology, microscopy hardware, and imaging software at imaging core facilities across the world. These grantees aim to increase interactions between biologists and technology experts. The NCI's R50 Research Specialist Award mechanism is an important experiment in this area as well.

Radical collaboration succeeds when investigators sense a near-absence of friction in their inter-institutional work. Investing in the creation of master templates for clear operational agreements can help quickly resolve issues with MTAs, expedite agreements between academia and biopharma, and align other inter-institutional obligations. It is likely that if major funders came together, they could craft holistic solutions to be adopted by nearly every institution to streamline work further. After all, we are all part of the same cancer-curing community.

## CONCLUDING REMARKS

Right now is a unique moment for the cancer community. We have the opportunity to take stock of the structural status of team science enterprises under way and optimize them, while accelerating the adoption of best practices.

There is work for all of us to do, and it has just begun. We hope that by articulating a shared ontology through these Hallmarks of Cancer Collaboration, it might spark others to further study and refine these concepts. Some solutions may drive reform in academia, while others may stimulate novel nonprofit structures (13). What's more, we call for a recurring series of interactions among the major international nonprofit and governmental funders of cancer team science. By working together—by emulating the same hallmarks we ask of scientific teams—funders can share best practices and accelerate systems change for the cancer community. Together, funders can increase the modularity and sustainability of projects, so that those projects with ambitious common visions—which often require timelines of 10 to 15 years minimum—receive either long-term funding or assistance in identifying and obtaining future funding.

The cancer community can accelerate change for the patients we serve. This is a radically collaborative future worth pursuing, a future to be proud of.
